# Nattokinase: An Oral Antithrombotic Agent for the Prevention of Cardiovascular Disease

**DOI:** 10.3390/ijms18030523

**Published:** 2017-02-28

**Authors:** Yunqi Weng, Jian Yao, Sawyer Sparks, Kevin Yueju Wang

**Affiliations:** 1 Emergency Department, The Affiliated Hospital of Qingdao University, Qingdao 266005, China; wengyunqi@126.com (Y.W.); yaojian2002@126.com (J.Y.); 2 Department of Natural Sciences, Northeastern State University at Broken Arrow, Broken Arrow, OK 74014, USA; sparks07@nsuok.edu

**Keywords:** nattokinase, oral, antithrombotic agent, cardiovascular disease, plant molecular farming, gene expression

## Abstract

Natto, a fermented soybean product, has been consumed as a traditional food in Japan for thousands of years. Nattokinase (NK), a potent blood-clot dissolving protein used for the treatment of cardiovascular diseases, is produced by the bacterium *Bacillus subtilis* during the fermentation of soybeans to produce Natto. NK has been extensively studied in Japan, Korea, and China. Recently, the fibrinolytic (anti-clotting) capacity of NK has been recognized by Western medicine. The National Science Foundation in the United States has investigated and evaluated the safety of NK. NK is currently undergoing a clinical trial study (Phase II) in the USA for atherothrombotic prevention. Multiple NK genes have been cloned, characterized, and produced in various expression system studies. Recombinant technology represents a promising approach for the production of NK with high purity for its use in antithrombotic applications. This review covers the history, benefit, safety, and production of NK. Opportunities for utilizing plant systems for the large-scale production of NK, or for the production of edible plants that can be used to provide oral delivery of NK without extraction and purification are also discussed.

## 1. Introduction

**Figure 1:**
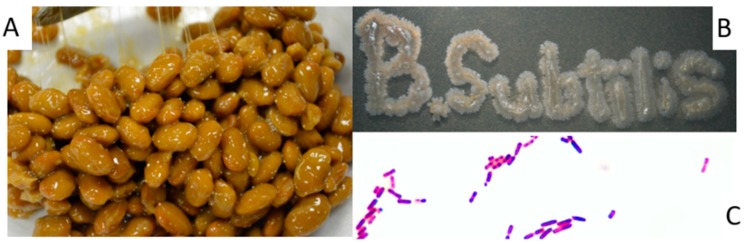
Natto—a traditional Japanese food produced from soybeans fermented by *Bacillus subtilis* (natto). (**A**) Natto-Fermented Soy Beans; (**B**) *B. subtilis* (natto) isolated from natto; (**C**) Micrograph of gram stained cells of *B. subtilis* (natto) (1000×).

**Figure 2:**
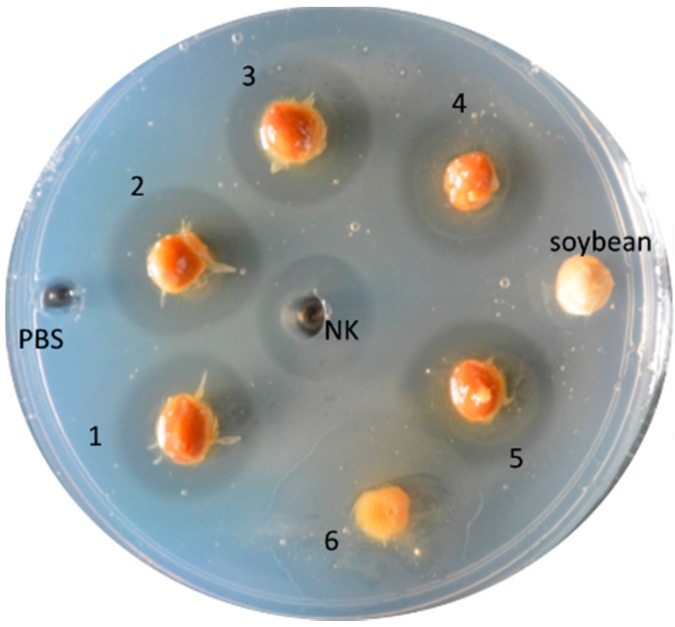
Natto and nattokinase can dissolve fibrin (semi-transparent halo ring). (1–5) Natto-fermented soy beans; (6) Slimy material characteristic of natto; (NK) Commercial nattokinase (100 µg) as a positive control; Non-fermented soybean and PBS (phosphate buffered saline) as negative controls.

Nattokinase (NK) is not related to any of the known kinases. NK is a serine protease purified and extracted from natto (Figure 1A), a traditional Japanese food produced from the fermentation of soybeans with the bacterium, *Bacillus subtilis* (natto) (Figure 1B,C)*.* Natto is regarded as a fibrinolytic miracle food (Figure 2). In 1980, Hiroyuki Sumi, a Japanese researcher at the Chicago University Medical School, discovered that natto can dissolve artificial fibrin [1]. Sumi and his team extracted an enzyme from natto that not only degraded fibrin but also a plasmin substrate. He named this novel, fibrinolytic enzyme “nattokinase” [1].

**Figure 3:**
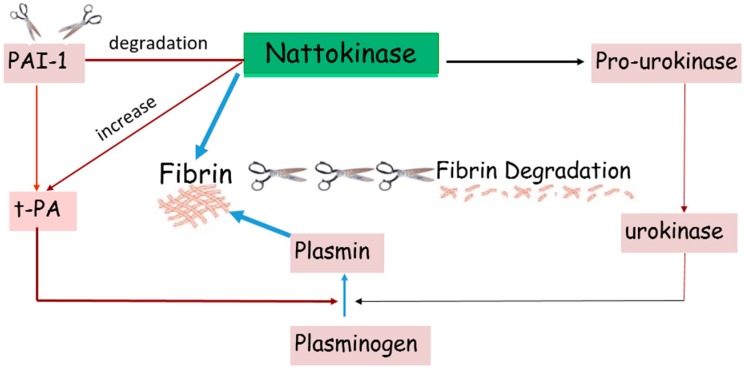
Mechanism of Action. Nattokinase dissolves blood clots by directly hydrolyzing fibrin and plasmin substrate. It converts endogenous prourokinase to urokinase (uPA). It also degrades plasminogen activator inhibitor (PAI-1) and increases the level of tissue plasminogen activator (t-PA).

NK can break down blood clots by directly hydrolyzing fibrin and plasmin substrate, converts endogenous prourokinase to urokinase (uPA), degrades PAI-1 (plasminogen activator inhibitor-1), and increases tissue plasminogen activator (t-PA) which supports fibrinolytic activity (Figure 3: Mechanism of action) [2]. Unlike common fibrinolytic proteases, such as t-PA and uPA, which can produce various side effects such as bleeding, NK exhibits little to no side effects. Studies also indicate that an oral administration of NK can be absorbed by the intestinal tract [3,4]. NK exhibits strong fibrinolytic activity after intraduodenal absorption. These characteristics make NK a versatile and potent fibrinolytic enzyme that can be used to combat blood clots.

## 2. Benefits of Nattokinase

Nattokinase is considered to be a safe, powerful, low cost, and all-natural supplement for the treatment of heart and cardiovascular disease [5,6,7]. Animal [3,4,8] and human trials [9,10,11] have demonstrated that NK provides support to the circulatory system by thinning the blood and dissolving blood clots. When dogs were orally administered four NK capsules (2000 FU/capsule), chemically-induced thrombi in the major leg vein were completely dissolved within five hours and normal blood flood was restored [8]. A rat model of thrombosis in the common carotid artery also demonstrated that NK-treated rats recovered 62% of arterial blood flow. NK exhibited considerably stronger thrombolytic activity than the fibrinogenolytic and fibrinolytic enzymes, plasmin, or elastase; which restored 15% and 0% of blood blow in the rat carotid artery, respectively [3].

NK was reported to have an effect on both oxidative injury-mediated arterial thrombosis [12,13] and inflammation-induced venal thrombosis [14]. When ferric chloride (FeCl_3_) was administered to the injured arteries, it resulted in oxidative thrombosis and platelet adhesion. After treatment with NK, however, thrombus formation and platelet aggregation were inhibited. The effects of NK are similar to the well-known blood thinner, aspirin [13]. Unlike aspirin, which often triggers bleeding or gastric ulcers, NK improves blood flow without any adverse effects. κ-Carrageenan-induced inflammatory thrombi formation in rat tails was used to examine the effect of NK [14]. Twelve hours after gavage administration of NK, higher levels of fibrin degradation product (FDP) fragments and d-dimers were detected in blood samples. A greater than 50% decrease in thrombosis was observed in the blood vessels of the rat tail by biopsy analysis.

Elevated levels of factor VII and VIII are associated with greater risk of cardiovascular disease due to the potential of these factors to trigger a blood coagulation cascade. In a human trial, three groups (healthy volunteers, patients with cardiovascular risk factors, and patients undergoing dialysis) were orally administered two capsules of NK (2000 FU/capsule) on a daily basis. After two months, a significant and similar decrease in factor VII, factor VIII, and fibrinogen was observed in all of the groups. No adverse effects were detected during the two-month trial and heart rate, body weight, and uric acid levels remained stable [15]. 

Nattokinase has a strong ability to breakdown thrombi and fibrin. Even a single dose of NK has been reported to result in fibrinolysis via the cleavage of cross-linked fibrin [10]. In that study, 12 healthy, young males were randomly administered a single capsule of NK (2000 FU). The antithrombin concentration in their blood increased significantly two hours after the oral consumption of the NK capsule. FDP fragments and d-dimers were observed four and six hours after NK administration, respectively, and factor VIII activity declined four hours after NK ingestion. The results of this study indicated that multiple different pathways may be involved in NK fibrinolysis and anti-coagulation activity.

Both NK and lumbrokinase (derived from earthworms), unlike most proteins, are more resistant to the highly acidic gastric fluids in the stomach and can be absorbed in the later sections of the digestive tract. In 1995, Fujita and colleagues demonstrated that NK could be absorbed from the rat intestinal tract in an intact form and degraded fibrinogen in plasma blood samples [3]. Subsequently, in 2013, a research team in the United States detected intact NK in the serum of healthy humans after they were administrated a single, oral dose of NK (2000 FU/100 mg) in a capsule [16]. Other studies have also shown that oral administration of NK can enhance fibrinolytic activity in plasma [3,16]. The mechanism by which NK is transported from the digestive tract into the circulatory system still needs to be elucidated. NK can resist high temperature (50 °C) and pH (to 10), which certainly contributes to the ability of this enzyme to remain intact in the gastrointestinal tract [6]. 

**Figure 4:**
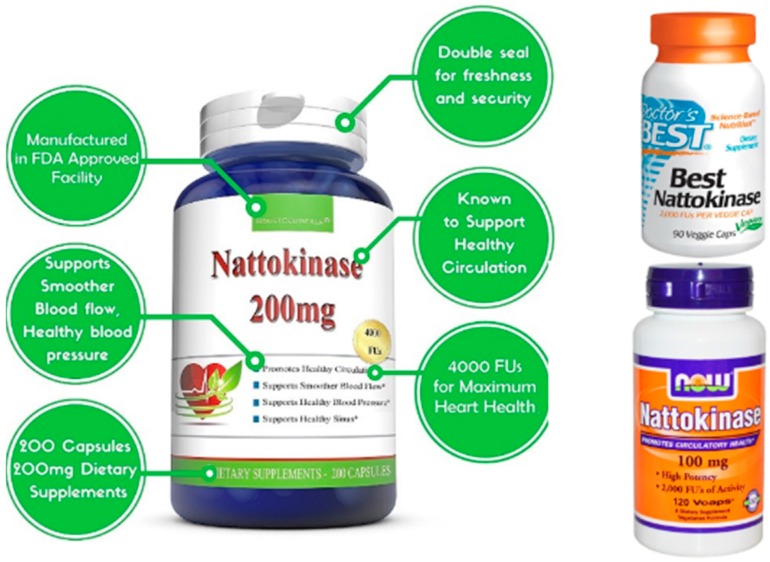
Nattokinase products (Pictures obtained from company websites).

At present, commercial NK products (Figure 4) are widely-used in Japan, China, Korea, European Union Countries, Canada, and the United States as a food supplement to thin blood, prevent blood clots, and improve blood circulation. Studies also indicate that NK can ameliorate other diseases such as hypertension [4], stroke [17], Alzheimer’s disease [18], and atherosclerosis [19]. The potential of using NK to decrease atherothrombotic risk and slow the progression of atherosclerosis as well as cognitive decline is currently being assessed in a Phase II clinical trial (ClinicalTrials.gov Identifier: NCT02080520, 28 May 2015).

## 3. Nattokinase Safety Assessment

Natto (Figure 1A), a soybean product fermented by the bacterium, *Bacillus subtilis* (natto), has been consumed as a traditional food in Japan for over a thousand years. Reports have suggested that natto contributes significantly to the longevity of Japanese people [1,5]. The accumulation of lipofusin (age pigment) is considered a hallmark of aging. In this regard, natto extract was reported to delay lipofusin accumulation in the nematode, *Caenorhabditis elegans*. The lifespan of *C. elegans* was also significantly prolonged by the Natto extract [20].

Although no adverse sides effects have been observed from the consumption of NK in various human trials, including clinical trials, the safety profile for NK, including the effect of repeat doses, acute toxicity, and genotoxicity, still needs to be thoroughly addressed. Comprehensive safety data, assembled under Good Laboratory Practice (GLP)-compliant studies and reported in 2016, indicated that neither clastogenic nor mutagenic activity was observed in vitro after NK treatment [11].

*B. subtilis* (Figure 1B,C), the bacterium responsible for the production of natto and the synthesis of NK, is not a pathogenic bacterium. The inoculation of rats with *B. subtilis* (1.51 × 10^9^ CFU/mL) derived from natto did not produce any signs of toxicity. Fourteen days after the treatment, no remaining viable *B. subtilis* cells were observed in the lungs, liver, brain, or kidneys by histopathological examination. No significant adverse signs or mortality were observed in an acute toxicity study within a 14-day study period when rats were gavage administrated a single dose of NK (2000 mg/kg). When rats were repetitively given single daily dose of NK (1000 mg/kg) for 90 days, no abnormal clinical observations were detected relative to control groups.

In human clinical studies, no-adverse-effect-level (NOAEL) was found when healthy human volunteers orally consumed NK (10 mg/kg) daily for 28 days [11]. Participants in the study exhibited no significant changes in their urine, blood pressure, or pulse. The collective data found in toxicity studies has provided a robust safety assessment for NK usage to both regulatory agencies and pharmaceutical companies. Presently, the recommended usage for NK is for two capsules (100 mg/capsule) daily. This dosage of NK has raised very low toxicological concerns based on the previously published safety studies.

## 4. Production and Purification of Nattokinase

The fermented soybean product, natto, is the main source for obtaining purified NK. A similar enzyme has been extracted from other fermented soybean-based foods, such as Thai thua nao [21], Chinese douchi [22], and Korean doen-jang [23].

The traditional process of fermenting soybeans to make natto is simple and straightforward, and can be easily done at home. *Bacillus subtilis* (natto) is the starter used to make natto, commercially and at home. *B. subtilis* (natto) can maintain activity at a pH of 6–12 and resist high temperatures up to 60 °C [24,25]. The *B. subtilis* strains present in current commercial NK products can maintain viability and metabolic activity at room temperature for at least six months. Cooked soybeans are inoculated with *B. subtilis* (natto) and incubated at room temperature to ferment for at least 24 h until the beans are covered with a viscous and sticky substance produced by the bacterium and consisting of glutamic acid polymers (Figure 1A). Commercial NK production practices optimize the fermentation conditions to maximize the yield of NK produced by *B. subtilis* (natto), and include optimal temperature, pH, and fermentation time [26,27]. A variety of nutrients, such as glycerol, yeast extract, soy peptone, or shrimp shell powder have been examined for their ability to increase NK yield [28,29,30]. The optimal feed strategy used in in fed-batch fermentation methods has significantly enhanced NK production, relative to yields obtained by batch fermentation [31].

Compared to the simple fermentation process, downstream extraction and purification of NK from natto slurry is difficult and inefficient. Several steps are required, including homogenization with an organic solvent, salting out the proteins, protein ion-exchange chromatography and dialysis, etc. Low NK activity recovery from these involved processes have driven researchers to investigate more inexpensive, rapid, and efficient techniques for NK purification [24,32,33]. Garg and Thorat [34] developed a three phase partitioning (TPP) technique to purify NK by combining t-butanol (1.5× to crude extract) and ammonium sulphate (30% *w*/*v*) to precipitate the NK protein. The desired results were obtained using an optimal pH (8.0) and temperature (37 °C). NK activity varies, however, when different purification methods are used. In addition, there is also potential for the retention of excessive byproducts in the final product than can cause an allergenic reaction [35].

The mild odor and stringy texture of natto is a major drawback to its use as a common food. Outside of Japan, NK is generally consumed orally in capsules typically made of vegetable-based materials for vegetarians. The presence of impurities in current NK products, however, prevents their common use as therapeutic medicine for thrombosis. Current NK products have raised concerns in the Federal Drug Administration (FDA) due to the requirement of high-levels of purity in single component entities. Thus, recombinant technologies have been explored to increase the quantities and purity of the NK being produced.

## 5. Analysis of the Nattokinase Gene and Protein

**Figure 5:**
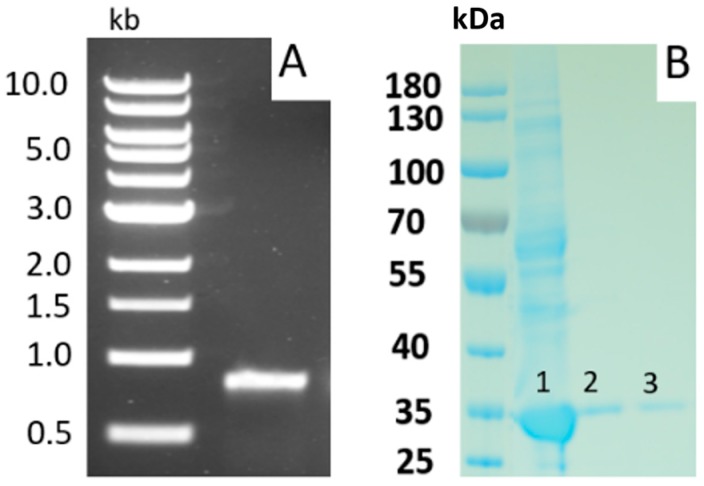
NK gene product and insoluble (inclusion-body) NK protein in *Escherichia coli*. (**A**) PCR-derived NK gene product from *B. subtilis* (natto); (**B**) Lane 1: NK protein present in crude medium extract; Lane 2 and 3: NK protein purified using a Ni-NTA (nickel-charged affinity nitrilotriacetic acid) column.

Nattokinase is encoded by the *aprN* gene, which was first cloned and sequenced from *B. subtilis* (natto) [36]. The full length polypeptide contains a 29-residue signal peptide which directs protein secretion through the cell membrane, and a 77-residue propeptide which plays a crucial role as an intramolecular chaperone during the protein folding process; resulting in a 275-residue, mature and functional NK peptide. The partial length of *aprN* gene is 0.8 kb in length (Figure 5A) and the mature peptide has a molecular weight of 27.7 kDa (Figure 5B). Since the amino acid sequence of NK is almost identical (99.3% homologous) to Subtilisin E, NK was also considered as a member of the subtilisin serine protease family. Unlike subtilisin, however, NK has a very specific affinity for fibrin degradation [37]. The D^32^-H^64^-S^221^ motif and N^155^ function as the catalytic triad and oxyanion hole of NK, respectively [38,39]. These sites are critical for the protease hydrolysis process. Based on the crystal structure of Subtilisin E, a 3D structural model of NK was constructed [38]. It indicated that the overall active centers are negatively charged, suggesting that NK is more specific for positive-charged substrates. 3D model predicted that the mechanism of NK was induced by attacking of hydroxyl rich in catalytic environment and locating of S^221^ [38]. Site-directed mutagenesis and molecular dynamics simulation of a 3D model revealed that hydrogen bonds formed between residues, S^33^, D^60^, S^62^, and T^220^ stabilize the transition state of the hydrolysis reaction. S^125^, L^126^, and G^127^ serve as substrate binding sites. Three residues of S3 binding sites, G^100^, S^101^, and L^126^ are responsible for fibrinolytic activity while they moderately affected substrate specificity [40].

**Figure 6:**
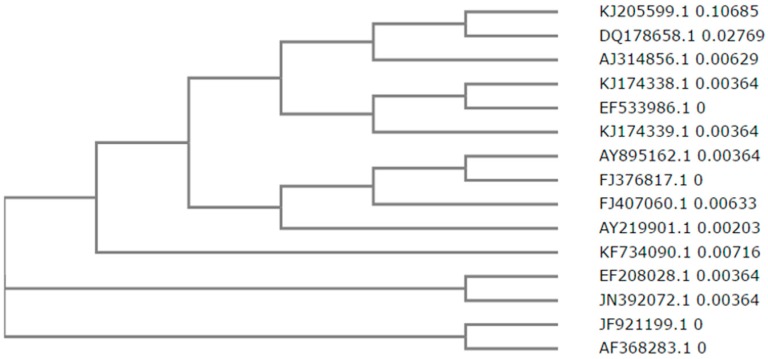
Phylogeny of NK sequences. Predicted NK protein sequences were generated using DNA to PROTEIN software (available at: http://web.expasy.org/translate/). Sequence analysis, multiple sequence alignment, and phylogenetic analysis were conducted using ClustalW2 (available at: http://www.ebi.ac.uk/Tools/msa/clustalw2/) software. A total of 16 NK gene sequences were deposited in the NCBI Genbank. Fifteen mature protein sequences were subjected to a phylogenetic analysis. HM068963.1 is only represented by a partial sequence and as a result, was not included in the phylogenetic analysis.

In addition to *B. subtilis* strains, researchers have isolated NK from marine organisms [41] and *Pseudomonas* sp. [42]. Sixteen NK gene sequences have been identified from various *B. subtilis* strains and their sequences are available in the NCBI GenBank. Amino acid sequence alignment of mature NKs demonstrates that they are highly homologous to each other; with some of the protein sequences possessing 100% identity. For example, the amino acid sequence of AF368283.1 and JF921199.1 are 100% identical. In addition, JN302072 and EF20828.1 also share the same amino acid sequences (Figure 6).

## 6. Recombinant Nattokinase Production via Genetic Engineering

In order to increase NK yields and simplify the downstream purification process, the NK gene has been cloned and expressed in various microbial host systems, including *Escherichia coli* [43,44,45], *B. subtilis* [46,47,48], and *Lactococcus lactis* [49]. *E. coli* has been extensively investigated as the easiest and cheapest host system to produce recombinant NK. Although NK can be expressed in *E. coli*, large amounts of recombinant protein aggregate results in the formation of insoluble and inactive inclusion bodies [43,44]. Recovery of bioactive NK from inclusion bodies is challenging. Most of the protein is lost during solubilization and refolding of the protein contained in the inclusion body is challenging. Liang et al. (2007) isolated an NK gene from Chinese douchi and fused it with a periplasmic secretion signal, PelB, and a native NK signal peptide [44]. Active NK was successfully expressed in *E. coli*, however, the fibrinolytic activity of the secreted, recombinant NK was considerably lower than NK isolated from natto. *B. subtilis* is also an attractive host for the production of recombinant NK since it has the capacity to produce secretory proteins. The –10 element (TACAAT) of the NK (*PaprN*) promoter was substituted with the consensus –10 region (TATAAT) and successfully enhanced NK expression (643 mg/L) in recombinant *B. subtilis* [50]. *B. subtilis* itself, however, produces a substantial number and quantity of native extracellular proteases which can hydrolyze recombinant proteins. In this regard, an extracellular-protease-deficient strain of *B. subtilis* has been used to produce enhanced levels of NK [51]. In that study, the NK gene was expressed under the control of the *acoA* promoter in *B. subtilis* WB800, a strain that lacks eight extracellular proteases. Although a high yield of 600 mg/L NK was achieved, the technique was not suitable for large-scale industrial application which requires the production of g/L to be economically feasible.

The use of eukaryotic expression systems for the production of NK have also been explored. A modified Bac-to-Bac^®^ baculovirus expression system was used to express and produce active soluble NK in *Spodoptera frugiperda* insect cells [52]. The high production costs and longer duration needed for expression (~two weeks) do not support the use of insect cells as an NK production system. A yeast expression system was also investigated as an NK production system [53]. In contrast to *E. coli* systems, yeast is able to perform post-translational modifications and possess the molecular machinery needed to fold recombinant proteins. The yeast, *Pichia pastoris*, in combination with a methanol-inducible promoter, has the ability to achieve a high density level during fermentation and can produce large quantities of recombinant protein. A low NK yield, however, was reported using a *P. pastoris* expression system; but the NK produced did exhibit fibrinolytic activity [53]. Methanol oxidation can induce cell death, and methanol also raises combustion concerns in large-scale industrial applications. Therefore, utilizing methanol to induce protein expression significantly limits the use of *P. pastoris* for the commercial production of NK.

## 7. Plants as Potential Factories for Nattokinase Production

Plant molecular farming (PMF), which utilizes plant systems for the production of recombinant human pharmaceutical proteins, has been investigated for over 30 years and is becoming an attractive alternative for the production of recombinant proteins [54,55,56]. Various difficulties—such as low yield, restrictive biosafety regulations, pollen contamination, and downstream processing challenges—have hindered its practical application [55,57]. The rapid development of genetic engineering technology, facilitated by bioinformatic, proteomic, and genomic advances are paving a pathway from the laboratory to the industrial production of recombinant proteins. In 2012, carrot-derived recombinant taliglucerase alfa (commercial name: ELELYSO™) became the first plant-based recombinant enzyme approved by the FDA to treat Gaucher’s disease [58]. In 2014, the Ebola virus epidemic attracted scientific attention worldwide and the need to develop a vaccine. The fatality rate from Ebola was nearly 50%, which resulted in approximately 11,000 deaths during that epidemic. No effective therapy was available for the treatment of Ebola, except ZMapp, a novel therapy utilizing a tobacco-expression system [59]. Seven Ebola patients achieved 100% recovery after being administered ZMapp [55]. ZMapp was granted a fast-track clinical trial by the FDA in September, 2015. In light of the two highlighted successes (ELELUSO and ZMapp), researchers have vigorously investigated the use of plants as factories for the production of human pharmaceuticals. Currently, over 20 plant-derived recombinant therapeutic proteins are in clinical trials, and several have received FDA approval [55].

Nattokinase is an ideal candidate for PMF. One of the major concerns in using PMF to produce pharmaceutical proteins, however, is plant glycosylation. Glycosylation of proteins is a normal occurrence and is used to regulate protein function during plant growth and development [60]. Unfortunately, when plant-derived therapeutic proteins are administered by injection, plant-specific glycan residues may invoke unwanted side effects in patients, such as immunogenicity [61,62]. Plant products, however, do not induce an immunogenic response when they are consumed through the digestive system. Presently, people consume genetically modified food on a regular basis. Since NK is mainly administered orally, using transgenic plants to produce NK would not face problems associated with immunogenic responses, and may find greater acceptance due to the benefits of the recombinant protein on human health.

**Figure 7:**
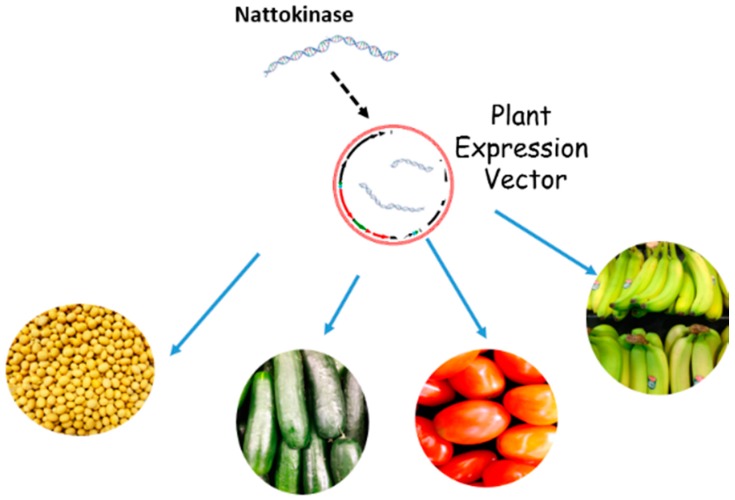
Plants as potential factories for NK production.

Using plant expression platforms for NK production could significantly reduce production costs (Figure 7). The expenses associated with the use of plant systems is very low, representing 0.1% to 10% of the cost associated with bacterial or other eukaryotic cell expression systems [63,64]. There are a variety of ways to produce NK in plant systems. A rapid method is the utilization of a transient expression system, which can produce high levels of recombinant protein within a relatively short period of time (four to seven days) [65,66,67,68,69]. Various virus-based transient vectors have been studied for use in PMF systems. The bean yellow dwarf virus (BeYDV)-based DNA replicon system, developed in the laboratory of Hugh Mason, is one of the well-known systems [70]. The BeYDV system produced 0.5 mg/g of Ebola antibody (ZMapp) in tobacco leaves [71]. In 2016, the BeYDV vector was optimized by modifying its 5′ and 3′ untranslated regions. Use of the optimized vector significantly enhanced the yield of recombinant protein (1.8 mg/L) in *Nicotiana benthamiana* leaves, which represented the highest yields of ZMapp ever reported [72]. Wang and Lauren (2014) successfully expressed NK in tobacco leaves utilizing the BeYDV system [73]. The plant-derived soluble NK produced using this system dissolved fibrin and blood clots in vitro. The high levels of recombinant NK produced in this system, however, also caused leaf necrosis. We have also expressed other blood-clot dissolving proteases, such as lumbrokinase, human tissue plasminogen activator (t-PA), and vampire bat plasminogen activator (DSPAs) in tobacco leaves [55]. In addition, all of the manufactured proteases caused plant cell death; resulting in leaf necrosis. The role of proteases in programmed cell death in animal cells is well known [74] and it is plausible that plant proteases may have a similar role.

Transient expression is relatively straightforward and scalable. Recombinant protein, however, has to be harvested in a timely manner; otherwise it is subject to proteolysis. Another approach to transient expression that overcomes the problem of proteolysis, is to express the recombinant protein—such as NK—in plant seeds. Recombinant proteins produced in this manner can be stored for long periods of time (up to three years), and are easy to harvest and transport. Seed-based systems have been investigated in rice [75], corn [76], *Arabidopsis* [77], tobacco [78], and soybean [79]. In transgenic rice seeds, the yield of recombinant human serum albumin is 10.58% of the total soluble protein (TSP) [75]. Cellobiohydrolase II (Cel6A) targeted to maize endosperm, represents 30% of seed TSP. Recombinant protein yield in soybean can reach 4% of TSP [80]. Since natural NK is produced from fermented soybeans, it represents an ideal platform for recombinant NK production. Soy formulations—such as soy milk, powder, or flour—are regularly fed to infants with little or no side effects. It is also possible to use soy formulation technology for NK application, which would require little to no downstream purification of recombinant NK from transgenic soybean seeds. We have expressed NK in tobacco seeds utilizing a seed-specific promoter (*Phas*). Seed-derived NK has also been demonstrated to dissolve fibrin (manuscript in preparation).

Downstream processing and purification of PMF-derived protein can be costly due to the presence of the large amounts of endogenous proteins, phenolic compounds, alkaline agents, lignin, and waxes produced by plants. Downstream processing costs have been estimated to represent 80%–90% of the total PMF production costs. NK, however, could be produced in edible plants, such as cucumber or tomato, with unprocessed plant material serving as the agent for oral delivery of the recombinant protein. This would eliminate expensive downstream extraction and purification costs. Another advantage of producing NK in plants is that plant cell walls, which are composed of complex carbohydrates, can potentially encapsulate the recombinant product and prolong enzyme activity as the protein passes through the gastrointestinal tract. As a result, NK activity would be protected before it enters into the circulatory system.

## 8. Conclusions

Nattokinase exhibits exceptionally potent fibrinolytic activity. Natto, a soybean product fermented by *B. subtilis* (natto) and rich in NK, has been served as a traditional food in Asia for hundreds of years and has recently garnered increased interest in Western medicine. Various animal and human trials have demonstrated that NK improves blood circulation and helps decrease the risk of a variety of cardiovascular diseases without producing any adverse side effects. The unpleasant odor and texture of natto limits its utilization as a dietary nutriment. Costly and complicated extraction and purification processes have inhibited the general use of NK as a nutraceutical. Currently, NK is sold as a dietary supplement in the United States, Canada, and Europe. It is available as a powder contained within a vegetable-based capsule. Ongoing advances in genetic engineering are providing a promising future for the economically-viable, large-scale production of high-quality NK using recombinant gene technology. Among the available expression systems (*E. coli*, yeast, and animal cell), plant expression represents a promising alternative system for the production of NK for direct consumption or further downstream processing and purification. 

## Abbreviations

NK	nattokinase
PMF	plant molecular farming
BeYDV	bean yellow dwarf virus
TSP	total soluble protein
